# Four new mouse models of Duchenne muscular dystrophy with clinically relevant exon deletions in the human *DMD* gene

**DOI:** 10.1242/dmm.052875

**Published:** 2026-08-28

**Authors:** Maaike van Putten, Margot Linssen, Christa Tanganyika-de Winter, Conny M. Brouwers, Jill W. C. Claassens, Nisha Verwey, Max Walsh, Tiberiu Loredan Stan, Annemieke Aartsma-Rus, Peter Hohenstein

**Affiliations:** ^1^ Department of Human Genetics, Leiden University Medical Center, 2333 ZC Leiden, The Netherlands; ^2^ Transgenic Facility Leiden, Leiden University Medical Center, 2333 ZC Leiden, The Netherlands

**Keywords:** Duchenne muscular dystrophy, Mouse model, Genome editing, Antisense oligonucleotide, Exon skipping

## Abstract

Variant-specific therapeutic approaches, such as exon skipping or gene editing, hold promise for the treatment of Duchenne muscular dystrophy (DMD). Translatability of preclinical studies investigating these approaches could greatly be improved through the use of humanized mouse models, as these allow preclinical testing of human-specific sequences. We developed four novel humanized mouse models of DMD with a deletion of exon 44, 45, 51 or 53 in the human *DMD* gene, in a mouse dystrophin-negative background (*mdx* mouse; exon 23 nonsense mutation). Our optimized prescreening pipeline allowed us to do so very efficiently with the CRISPR-Cas9 technology. We confirmed either complete lack of dystrophin or expression of trace levels, which led to development of muscle pathology consisting of muscle fiber degeneration and regeneration, inflammation and fibrosis in young adult mice. Intramuscular treatment with vivo-morpholinos targeting a flanking exon induced exon skipping in the DMD strains, which restored the disrupted open reading frame and, subsequently, dystrophin expression. This validates these models as valuable tools for preclinical studies investigating human sequence-specific therapeutic approaches for DMD.

## Teaser

**Summary:** Humanized mouse models of Duchenne muscular dystrophy were created with deletions of exon 44, 45, 51 or 53 in the human *DMD* gene. These dystrophic models allow preclinical testing of human-specific dystrophin-restoring approaches.

## INTRODUCTION

Duchenne muscular dystrophy (DMD) is an X-linked, progressive, muscle-wasting disease that is caused by pathogenic variants in the dystrophin protein-encoding *DMD* gene (Muntoni et al., 2003). Normally, dystrophin protein connects the cytoskeleton of muscle fibers to its connective tissue surrounding, by connecting F-actin and β-dystroglycan with its N- and C-terminal domains, respectively. Loss of dystrophin protein makes muscle fibers vulnerable to contraction-induced damage, leading to chronic inflammation, failure to regenerate, and, eventually, replacement of muscle tissue by fibrotic and adipose tissues. Patients irreversibly lose muscle function; even with multidisciplinary care, they generally lose ambulation in their early teens, need assisted ventilation around the age of 20, and die in the second to fourth decade of life due to respiratory or heart failure (Duan et al., 2021).

Whereas the pathogenic variants carried by patients with DMD prevent production of functional dystrophins (Duan et al., 2021), variants that do not disrupt the open reading frame and are located in the center of the gene allow the production of partially functional dystrophin proteins (Duan et al., 2021). These variants cause the allelic disease called Becker muscular dystrophy (BMD). This disease generally has a later onset and a slower disease trajectory than DMD. For both DMD and BMD, most patients carry large deletions involving one or more exons, which cluster between exons 42 and 55 (Bladen et al., 2015).

Therapeutic approaches aiming to restore dystrophin for DMD are based on the discrepancy between out-of-frame variants observed in patients with DMD and in-frame variants found in patients with BMD. Exon skipping and gene editing approaches aim to restore the open reading frame for *DMD* transcripts at the RNA and DNA level, using antisense oligonucleotides (ASOs) and CRISPR/Cas9, respectively. These approaches are variant specific, as, based on the size and location of the variant, different exons need to be skipped to restore the reading frame (Niks and Aartsma-Rus, 2017; Roberts et al., 2023).

Animal models for DMD have been invaluable for studying disease pathology. For therapeutic development, mouse models have been essential (van Putten et al., 2020; Zaynitdinova et al., 2021). The most commonly used mouse model for DMD is the *mdx* mouse (Bulfield et al., 1984), and standard operating procedures for its use in preclinical studies have been developed (Willmann et al., 2011). This model carries a spontaneous nonsense mutation in exon 23 of the mouse *Dmd* gene (Sicinski et al., 1989). It originally arose on the C57BL/10ScSnJ background, but has since then been backcrossed to other backgrounds, including C57BL/6J and DBA/2J (Fukada et al., 2010).

**Fig. 1.**
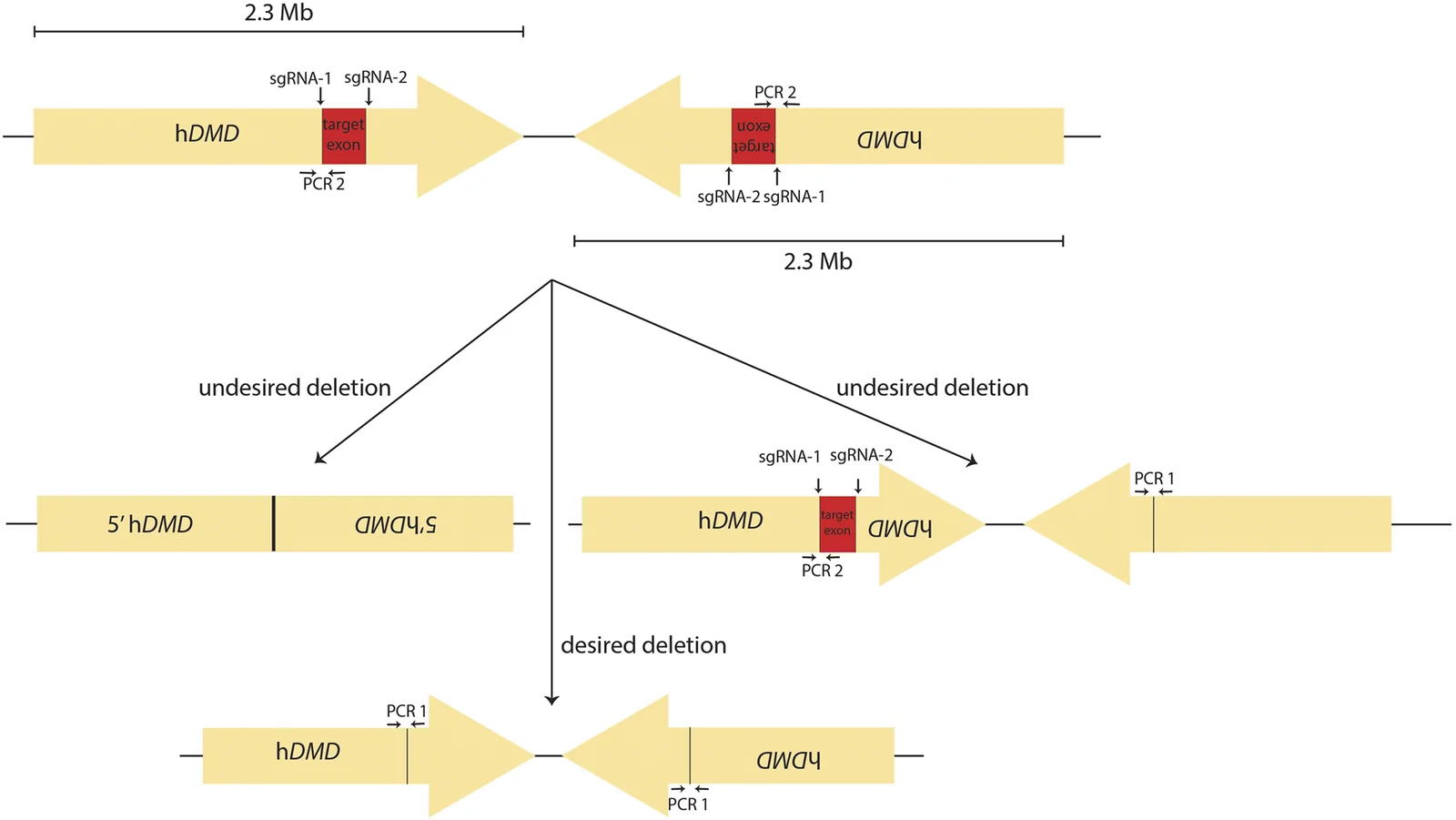
**The *hDMD* YAC transgene locus and prescreen PCR design for generating new exon deletion mutants.** Two PCR assays were sufficient to identify candidates for correct deletion of both copies of the target exon. PCR 1 gives a predictable fragment size after correct deletion by spanning the target exon; PCR 2 detects the retention of the target exon and the 5′ end of the human *DMD* (referred to as *hDMD*) gene. The hDMD yeast artificial chromosome (YAC) transgenic mouse has two copies of the *hDMD* YAC in a tail-to-tail configuration and scores negative for the predicted product size for PCR 1. Mice scoring positive for a PCR fragment of the predicted size for PCR 1 have lost at least one of the copies of the target exon and must have retained the 5′ half of both copies of the YAC. The mice were further tested for absence of PCR 2, which is specific for the undeleted target exons. Mice positive for PCR 1 and negative for PCR 2 were selected for further quality screening. sgRNA, single-guide RNA.

The *mdx* mouse has been used to provide proof of concept for the ability to restore dystrophin with exon skipping and gene editing approaches (Lu et al., 2003; Nelson et al., 2016). However, these studies focused on ASOs and guide RNAs targeting mouse dystrophin exon 23 and, therefore, were poorly translatable to the human situation. First, different ASOs and guide RNAs were required, as the mouse equivalents were not effective in humans owing to species-specific differences in sequences. Second, most patients with DMD have pathogenetic variants in a different region of the gene, and, as such, the dystrophin protein produced by the *mdx* mouse after exon skipping or gene editing differs from the ones that will be produced by the majority of patients. To allow testing of human-specific ASOs, a yeast artificial chromosome (YAC) transgenic mouse model carrying the complete 2.3 Mb human *DMD* (referred to as *hDMD*) locus was generated (’t Hoen et al., 2008). This model carried the *hDMD* gene on mouse chromosome 5. The expression of human dystrophin protein was capable of rescuing the *mdx* phenotype. To facilitate preclinical studies with human-specific ASOs or guide RNAs, models with pathogenic variants within the *hDMD* gene are required. We previously used transcription activator-like effector nucleases (TALENs) to delete human exon 52 from the *hDMD* gene as a first effort towards this goal (hDMDdel52/*mdx* strain) (Veltrop et al., 2018). In the course of validating this model, it was found that the *hDMD* YAC model carries two copies of the YAC in a tail-to-tail orientation, whereas the hDMDdel52/*mdx* model carries a partial deletion of exon 52 on both of the *hDMD* copies (Yavas et al., 2020). This realization explained why generating the hDMDdel52/*mdx* model had failed using homologous recombination. Furthermore, the tail-to-tail orientation severely complicated the generation of additional deletion models with genome editors, as there was a risk of deleting the target exon (e.g. exon 45) but also the entire content in between [exons 45-79 from both copies of the YAC (Fig. 1)].

Here, we optimized the procedure to make additional clinically relevant models from the hDMD/*mdx* mouse and used this to generate four new DMD mouse models carrying a deletion of exon 44, 45, 51 or 53 in the *hDMD* gene in the C57BL/6J/*mdx* background. We further demonstrate dystrophin restoration after ASO-induced exon skipping for each of the models to validate them for preclinical testing of human-specific therapies.

## RESULTS

The hDMD/*mdx* mouse model carries two tail-to-tail copies of the *hDMD* gene and remnants from the YAC in its integration site on mouse chromosome 5 (’t Hoen et al., 2008; Chey et al., 2024; Yavas et al., 2020). These complex genetics severely complicate the generation of exon deletions in the *hDMD* gene using genome editors (such as CRISPR/Cas9, TALENs or zinc finger nucleases). Using editors on each side of the target exon, a large deletion between editor target sites could be induced, resulting in the loss of the 3′ end (downstream of the target exon) of both copies (Fig. 1). We optimized the PCR-based prescreen of edited samples based on this knowledge by combining a PCR assay spanning the predicted deletion, to confirm the presence of the intended allele and retention of the region 3′ from the target, with a PCR assay with primers outside and inside the deletion to score for the absence of the target exon. The first assay scores positive for correct deletion of the target exon, but false positive if only one of the two copies of the target exon is deleted, while the second assay identifies these with a positive result. Combined, these two assays should efficiently identify the candidate clones or animals for further detailed testing (Fig. 1).

### Screening of the hDMDdel44/*mdx* model

**Fig. 2.**
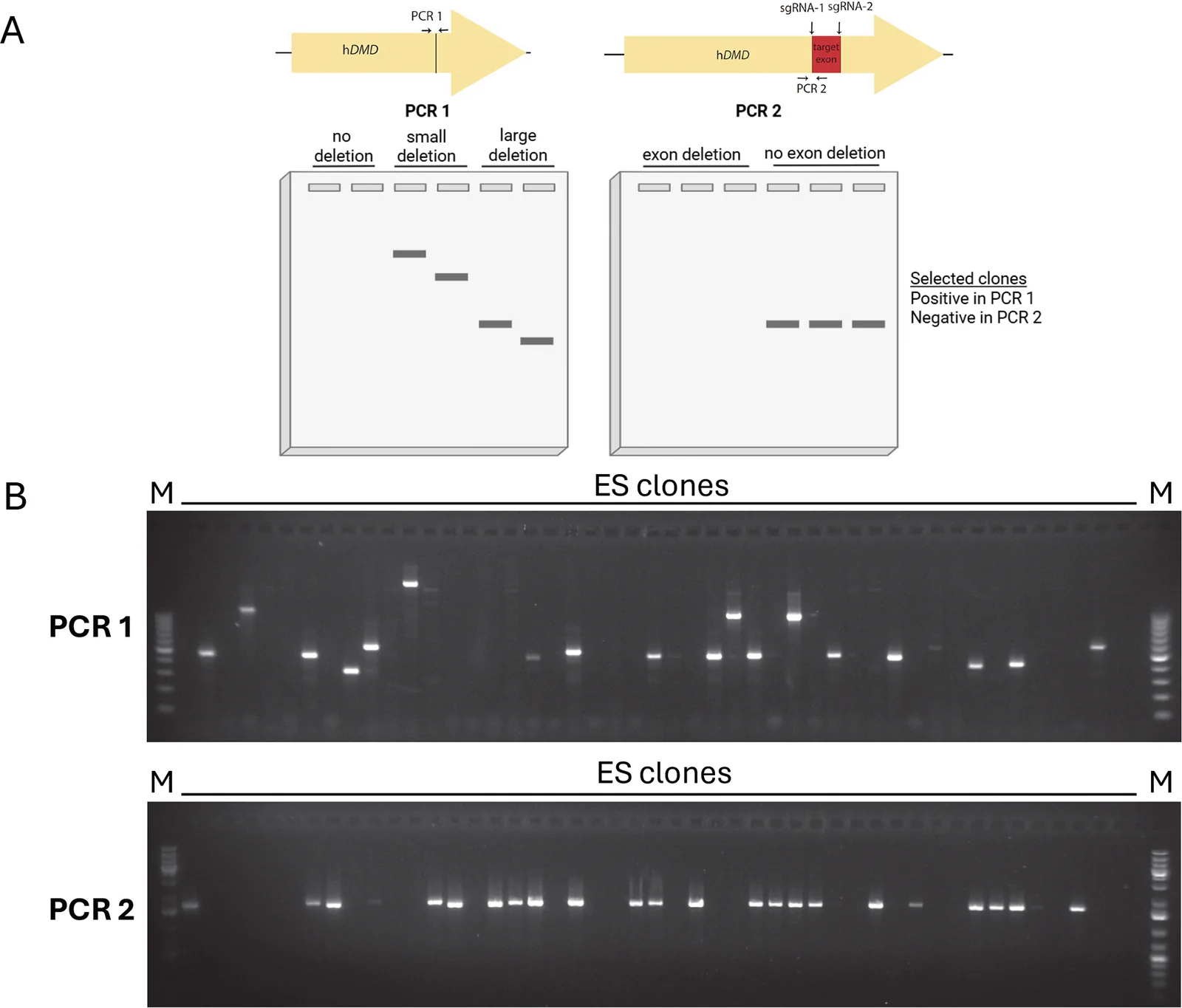
**Prescreen PCR of hDMD/*mdx* ES cells targeted for deletion of exon 44.** (A) Illustration of expected PCR product sizes for PCR 1, performed after transfection, which aimed to detect clones with a deletion of exon 44, in which the product size depends on the size of the deletion; and PCR 2, performed on clones positive in PCR 1, which aimed to check for the absence of the undeleted exon 44. (B) PCR 1: example of ES clones after transfection with CRISPR/Cas9 and sgRNAs designed to delete *hDMD* exon 44. The extension time of the PCR was chosen to be unable to amplify the 3.9 kb fragment of the undeleted allele, but to be efficient in amplification of fragments that were shorter owing to the deletion. PCR 2: example of PCR assay on del44-positive ES clones to detect any remaining exon 44. Clones that scored positive for this assay would have lost only one copy of the exon and were discarded. Only clones positive for PCR 1 (A) and negative for PCR 2 were tested further. ES, embryonic stem; M, 100 bp ladder.

**Fig. 3.**
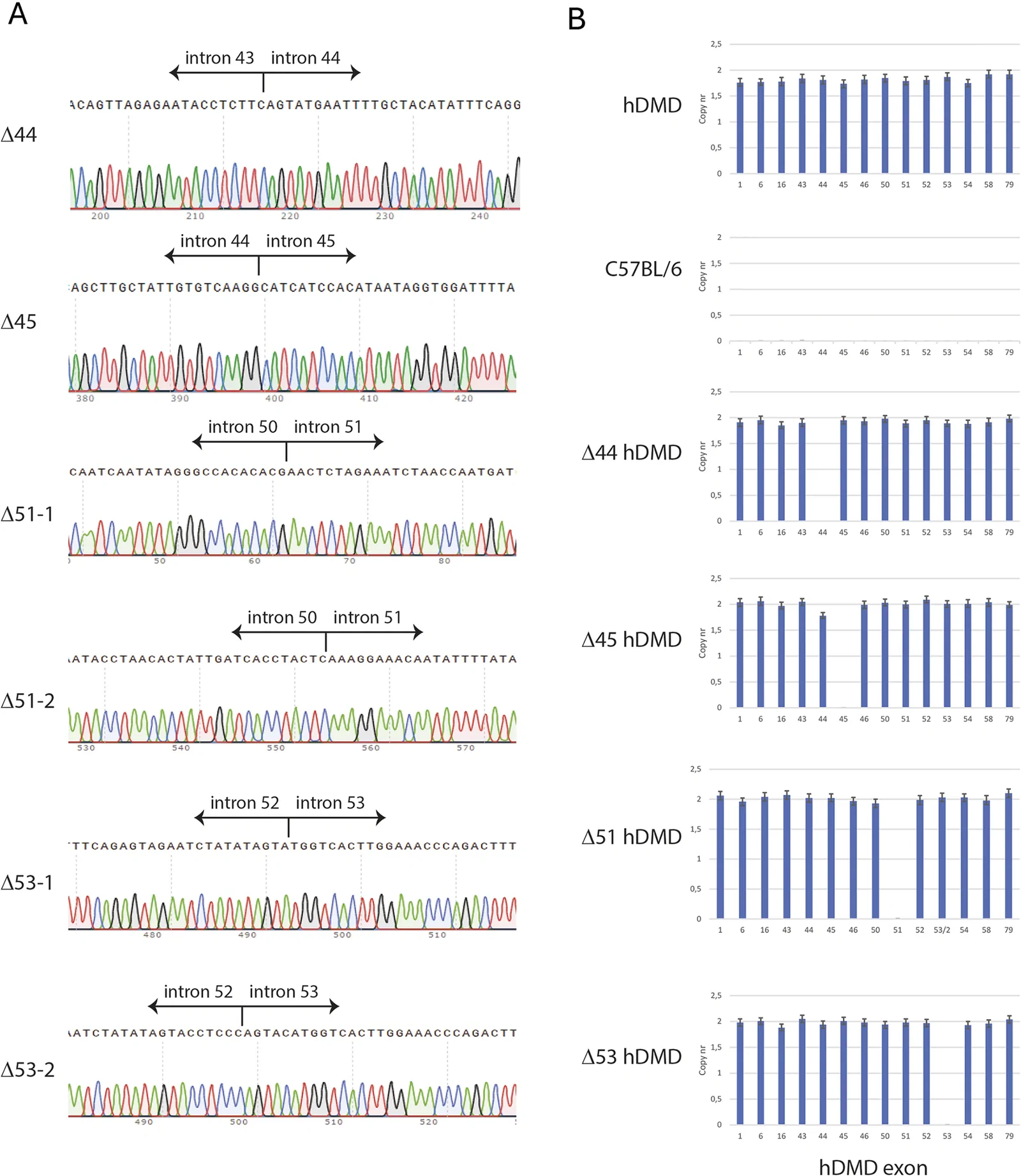
**Additional quality control for new hDMD models.** (A) Sanger sequencing of all mutant YAC alleles. Del51-1 and del51-2 are the two genetic variants observed in the founder animals; del53-1 and del53-2 are the two different deletions in the two copies of the YAC. (B) Copy number of mutant and control exons using digital droplet PCR.

We tested the revised prescreen by deleting exon 44 of the *hDMD* gene in embryonic stem (ES) cells that had previously been derived from the hDMD/*mdx* mouse model (Veltrop et al., 2013). CRISPR/Cas9 ribonucleoprotein (RNP) complexes targeting introns 43 and 44 were used to delete human exon 44 from these cells, and clones were tested with the new prescreen assays. We found an unexpected high percentage of candidate clones (14%) passing both steps of the prescreen (Fig. 2). Not every clone with a del44-positive PCR fragment was the same length, suggesting different deletions in different clones. This is to be expected, as non-homologues end-joining repair will not always give the same outcome and can be biased to certain deletions by microhomology-dependent repair. Selected clones were further expanded, retested and subjected to chromosome counts. A clone that passed the quality control criteria was used for blastocyst injection, chimera production and breeding for germline transmission to generate the hDMDdel44/*mdx* mouse model. Amplification and Sanger sequencing of the target region (Fig. 3A; Table S2) showed that, for the del44 model, indeed only one deletion could be detected, suggesting that both copies of the YAC had obtained exactly the same deletion.

### Screening of the hDMDdel45/*mdx*, hDMDdel51/*mdx* and hDMDdel53/*mdx* models

Encouraged by the specificity of our new prescreen workflow and the efficiency of correct targeting of human exon 44 in ES cells, we generated additional models with a deletion of exon 45, 51 or 53 directly in mouse zygotes. Where possible, we used homozygous hDMD/*mdx* males and wild-type C57BL/6J females to generate zygotes in which every embryo carried a single tail-to-tail *hDMD* insertion. Mutation-specific prescreen PCRs were used to identify candidate founder animals for the del45, del51 and del53 models.

Founder animals of the del45, del51 and del53 models were bred for germline transmission and mice hemizygous for two copies of the intended mutant YACs were readily obtained. Amplification and Sanger sequencing of the target region (Fig. 3A) showed that, for the del45 model, only one deletion could be detected, while for the del51 model, we initially only identified founder animals lacking one copy of the target exon, with the second copy still intact. We therefore selected a second set of single-guide RNAs to completely delete exon 51. These single-guide RNAs were located within the region already deleted in the first copy of the YAC, thus ensuring that the already mutant YAC could not be edited further. Mice carrying the single del51 allele were used to generate new zygotes, which were targeted with the new single-guide RNA set. In this second targeting round, we were successful in identifying founder animals lacking both copies of exon 51 (Fig. 3A).

For the del53 model, we initially obtained only mixed Sanger sequencing traces, suggesting two different deletions in the two copies of the YAC. Indeed, TOPO cloning of the PCR product and sequencing of individual clones could separate the two deletions (Fig. 3A).

### Copy number analysis of the new hDMD deletion models

To test the integrity of the remaining parts of the YAC alleles in each model, we performed digital droplet PCR (ddPCR) copy counts of selected exons spread over the totality of the *hDMD* gene, including the target exons and exons directly upstream and downstream from the target exons. This analysis showed the presence of two copies for each exon (as expected from the double YAC integration in the original model) except for the target exon in each new model, which was absent (Fig. 3B).

### Validation of a muscular dystrophy phenotype in the hDMD deletion models

**Fig. 4.**
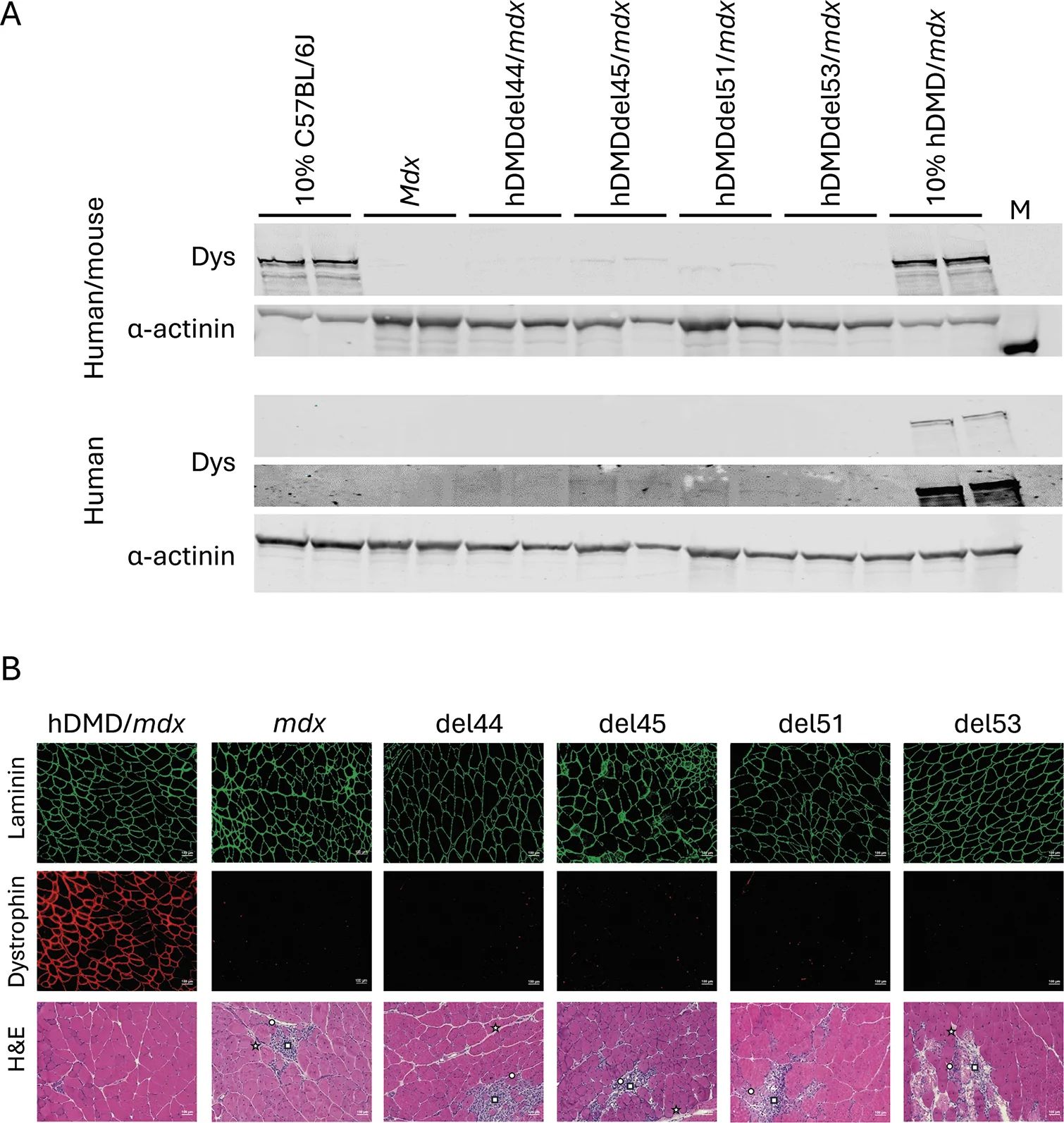
**Characterization of dystrophin expression and histopathology.** (A) Dystrophin expression [of either human/mouse (top; ab154168 primary antibody) or of human-specific origin (bottom; Mandys106 primary antibody, with a low and high exposure for dystrophin)] assessed by western blotting revealed that the hDMDdel44/*mdx* and hDMDdel53/*mdx* gastrocnemius completely lacked dystrophin expression, while traces of dystrophin were found in the hDMDdel45/*mdx* and hDMDdel51/*mdx* mice. α-actinin (ab72592 primary antibody) served as loading control. For each strain, protein samples of two individuals were loaded, except for the hDMDdel53/*mdx* strain, for which muscles of the left and right limb were obtained from the same individual. M, marker, with a band shown at 85 kDa. Both C57BL/6J and hDMD/*mdx* wild-type protein samples were included to validate the species-specific reactivity of the anti-dystrophin antibodies used. (B) Laminin (top row), dystrophin (middle row), and Hematoxylin and Eosin (H&E; bottom row) staining in the gastrocnemius of 8-week-old mice (utilizing ab15277 and sc-59854 as primary antibodies to stain for dystrophin and laminin, respectively). Barely detectable dystrophin positivity was observed in the hDMDdel51/*mdx* model. Histopathology shown consisted of degeneration and regeneration, inflammation and fibrosis, being indicated by circles, squares and asterisks, respectively. Scale bars: 100 μm.

The hDMD/*mdx* model and its deletion derivates are primarily of the C57BL/6J background. To reduce the amount of backcrosses required to generate a line with the *mdx* mutation, we crossed the models directly to the *mdx* line of the C57BL/6J background. The effects of the exon deletion on dystrophin expression in the novel models were assessed by western blot and immunofluorescence analyses of gastrocnemius muscles of 8-week-old males. As expected, the gastrocnemius of healthy hDMD/*mdx* mice expressed dystrophin of human origin at wild-type levels. Dystrophin expression in the hDMD deletion models was, however, lacking (hDMDdel44/*mdx* and hDMDdel53/*mdx*), or trace levels of dystrophin of human origin were observed (hDMDdel45/*mdx* and hDMDdel51/*mdx*) (Fig. 4A,B). The gastrocnemius of all novel deletion models was characterized by the classical histopathological hallmarks of the disease, comparable to those found in *mdx/*BL6 mice (van Putten et al., 2019) and consisting of muscle degeneration and regeneration, inflammation and fibrosis (Fig. 4B).

### Validation of the applicability of the hDMD deletion models for exon skipping

**Fig. 5.**
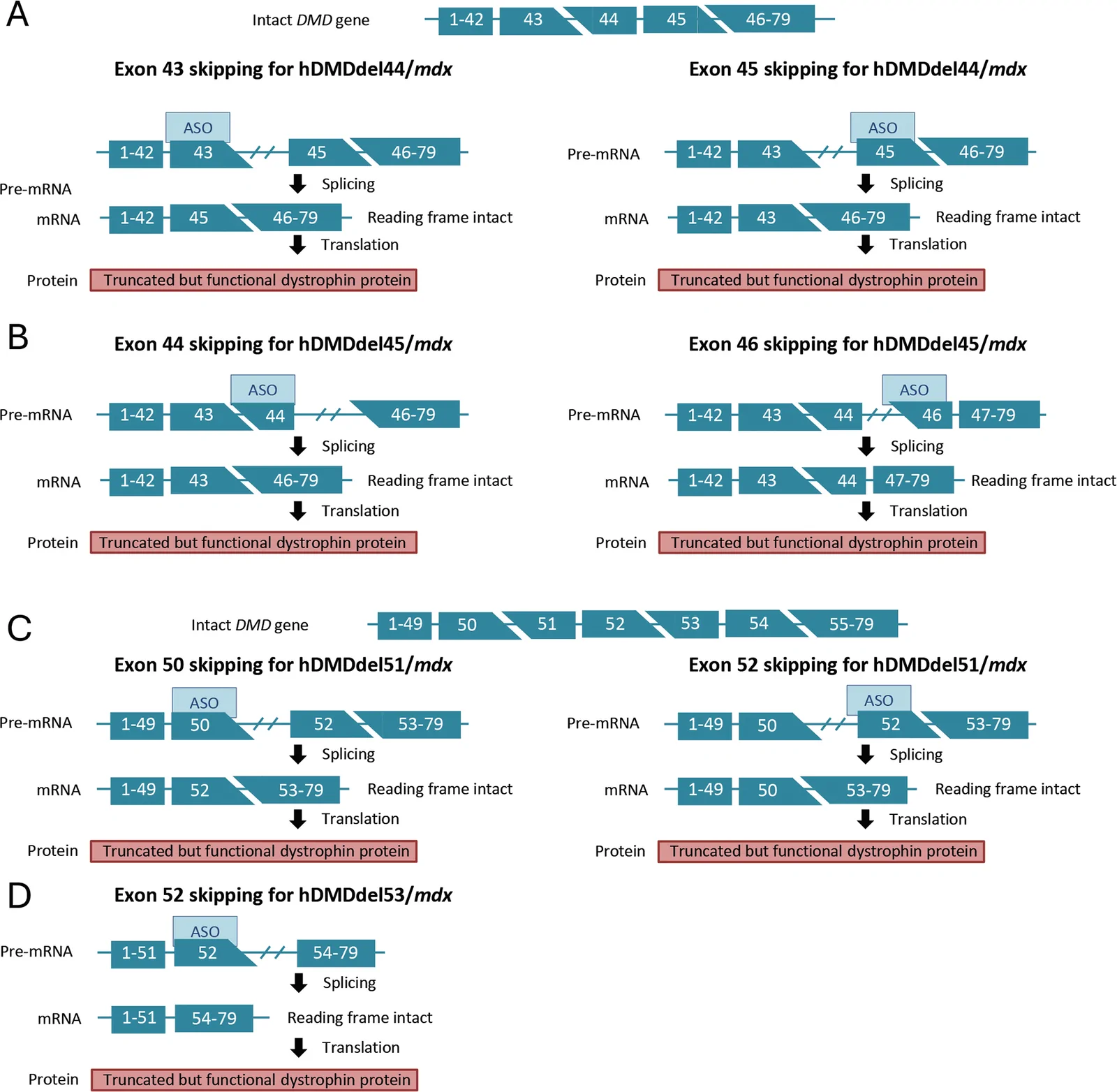
**Applicability of exon skipping to restore the reading frame in the humanized models.** (A) Skipping of either exon 43 (left) or 45 (right) restores the reading frame of the hDMDdel44/*mdx* mouse. (B) Skipping of either exon 44 (left) or 46 (right) can restore the reading frame of the hDMDdel45/*mdx* mouse. (C) Antisense oligonucleotides (ASOs) targeting exon 50 (left) or 52 (right) can be applicable for restoring the reading frame of the hDMDdel51/*mdx* mouse. (D) The reading frame of the hDMDdel53/*mdx* model can be restored by skipping of exon 52.

**Fig. 6.**
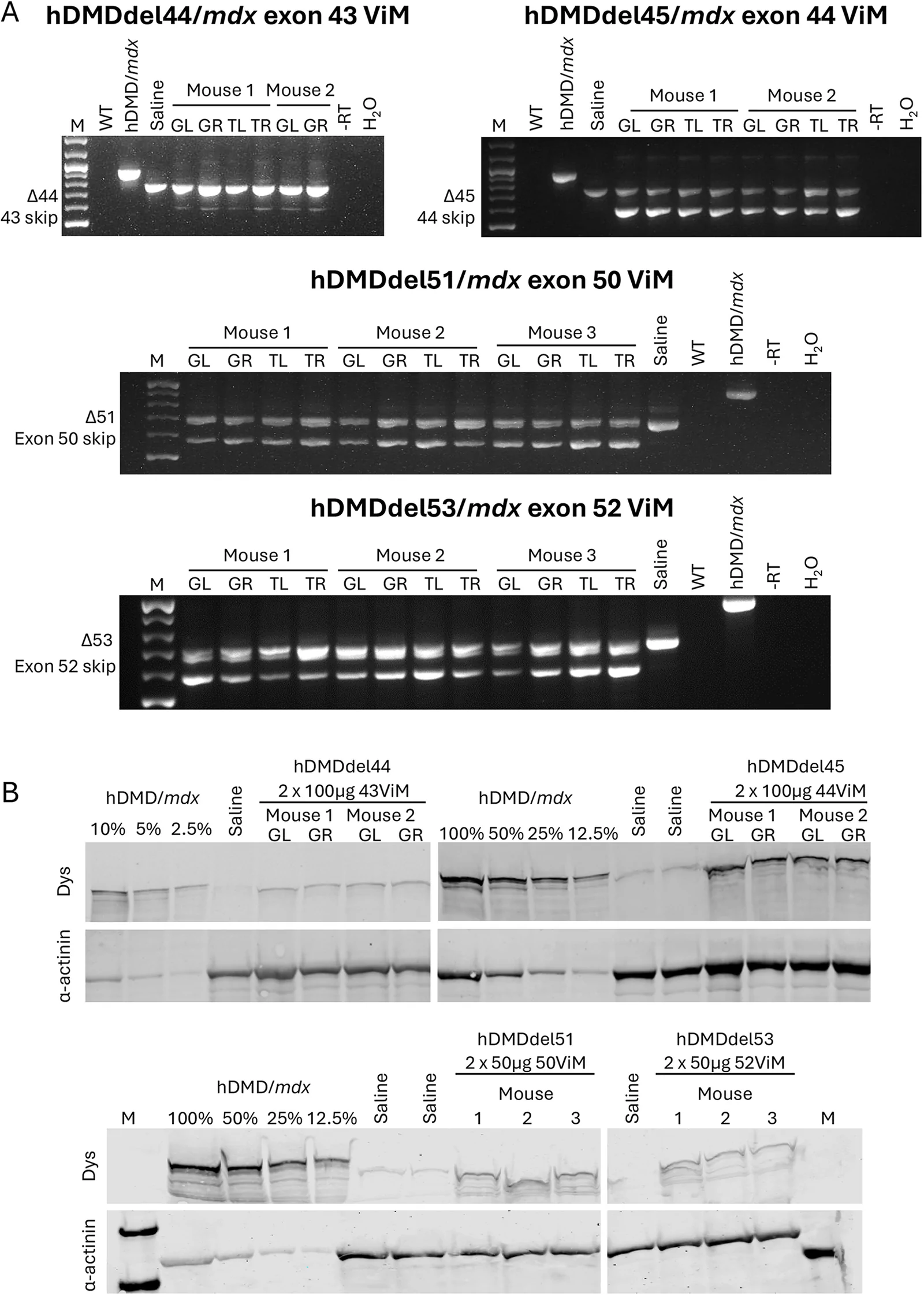
**ASO-mediated exon skipping restores dystrophin expression in the deletion models.** (A) A single human-specific PCR revealed skipping of exon 43, 44, 50 or 52 upon vivo-morpholino (ViM) injection of the right and left gastrocnemius and triceps muscles of either two or three hDMDdel44/*mdx*, hDMDdel45/*mdx*, hDMDdel51/*mdx* and hDMDdel53/*mdx* male mice. The upper (unedited) band corresponds to the PCR product only lacking the deleted exon, while the lower (skipped) band additionally lacks the nucleotides of the skipped exon. The double band is a cryptic splicing event, whereby exon 51 is spliced to part of intron 51 and then exon 52 (the part not deleted), which is then spliced to exon 53 (previously observed by Veltrop et al., 2018). GL, left gastrocnemius; GR, right gastrocnemius; M, 100 bp ladder. -RT, no reverse transcriptase; TL, left triceps; TR, right triceps; WT, C57BL/6J sample. (B) Western blot analyses utilizing the ab154168 (anti-dystrophin) and ab72592 (anti-α-actinin) primary antibodies confirmed that ASO-induced skipping of the respective exons restored the disrupted open reading frame and thereby dystrophin expression in all the deletion models. A reference series of several dilutions of a wild-type sample was taken as reference of the dystrophin levels restored. Analyses were conducted on one to two gastrocnemius muscles of two to three mice per strain, as shown in the figure.

To validate that exon skipping can restore dystrophin expression in these models, we designed ASOs targeting exon 43 (for the hDMDdel44/*mdx* model), exon 44 (for the hDMDdel45/*mdx* model), exon 50 (for the hDMDdel51/*mdx* model) and exon 52 (for the hDMDdel53/*mdx* model) (Fig. 5). Either 100 μg (exon 43 and 44) or 50 μg (exon 50 and 52) vivo-morpholino (ViM) was injected intramuscularly in the gastrocnemius and triceps of both limbs on two consecutive days. Two weeks after the last injection, muscles were isolated, and RNA and protein were isolated from muscle sections obtained from cryosections representative of the entire muscle. Skipping of the targeted exon was confirmed with reverse transcription PCR in each of the exon deletion models (Fig. 6A). As expected, restoration of the disrupted open reading frame induced expression of truncated dystrophin proteins as assessed by western blot analysis in gastrocnemius (Fig. 6B) and triceps (Fig. S1).

## DISCUSSION

Humanized mouse models are invaluable for preclinical testing of therapies that rely on the human DNA, RNA transcript or protein sequences of the causative mutated gene. We previously generated a humanized hDMDdel52/*mdx* model, which has since been widely used for preclinical studies (Van Deutekom et al., 2023; Engelbeen et al., 2023; Lim et al., 2022; Oppeneer et al., 2025a,b). However, generating this model was cumbersome, and, while doing so, we identified a double tail-to-tail integration of the human YAC in these mice (Yavas et al., 2020).

Here, we show that optimizing the prescreening strategy of genome-edited ES clones and founder animals based on this knowledge leads to the efficient generation of new humanized patient-specific mutant DMD mouse models. We generated four new models for the recurring deletions of exon 44, 45, 51 and 53. These models either completely lack dystrophin or express trace levels. This is in line with the trace dystrophin levels observed in patients with DMD amenable to e.g. exon 44 skipping and further increases the translational value of these models. We hypothesize that this phenomenon results from spontaneous exon skipping in some myofibers. The novel models all develop histopathology comparable to that found in the classic *mdx* model (van Putten et al., 2019). Currently, work is ongoing to fully characterize the natural disease history of these newly derived models (motor function, histological and molecular levels) throughout their lifespan. This will reveal whether there are actual differences between the models in disease development and severity and trace dystrophin levels and can further guide preclinical study design. Furthermore, we showed that ASO-mediated skipping of a flanking exon restores production of dystrophin, thus validating the models to be used to optimize mutation-specific therapies.

Together with the hDMDdel52/*mdx* model, these new models cover the top four most frequently observed single-exon deletions found in patients with DMD [exon 45 (4%), 51 (3%), 44 (3%) and 52 (3%)], and they could be utilized to test single-exon-skipping ASOs that would combinedly be applicable for over 50% of mutations (Bladen et al., 2015). It is challenging to give an exact number, as some mutations are amendable to skipping of the exon before or after the deletion. For example, patients with a mutation of exon 44 are amenable to skipping of either exon 43 or 45. Thermodynamic analysis of short peptides suggests that dystrophins lacking exon 44-45 are more stable than those lacking exon 43-44 (Ruszczak et al., 2009), suggesting that, for this deletion, exon 45 skipping may be preferred. This could also explain why lower levels of dystrophin restoration were observed in the hDMDdel44/*mdx* model after exon 43 skipping, although it is possible that this was also due to suboptimal ASO design.

With our optimized prescreening workflow, it would be straightforward to create additional patient-relevant preclinical mouse models for human sequence-specific DMD therapies. The two copies of the YAC in our humanized model do not complicate the use of these models for testing ASO-mediated exon skipping preclinically. In contrast, the double integration of the gene makes the protein expression levels of human origin in the mouse model more comparable to those of healthy control mice (’t Hoen et al., 2008). However, gene editing-based therapies could run into reduced targeting efficiency, comparable to what we experienced when generating these mutant models, and preclinical outcomes could therefore be underestimated. Recently, Chey et al. (2024) sequenced the complete double integration site of the YAC transgenes in our hDMD/*mdx* model and described guide RNAs that can be used to delete one copy of the YACs (Chey et al., 2024). This approach could be used to convert our mutant models into single-copy models as well, facilitating preclinical investigations on gene editing for DMD.

Taken together, we here present four novel dystrophic mouse models of DMD that allow for preclinical testing of human sequence-specific therapeutic approaches aimed at restoring dystrophin expression. Utilization of these models could greatly enhance translatability of preclinical findings to the clinic.

## MATERIALS AND METHODS

### Mouse lines

The following mouse lines were generated or used in this study: Tg(DMD)72Thoen/J (MGI:5578551); Dmd*mdx* (MGI:1856328); Tg(DMD*)del44Lumc (MGI:7408337); Tg(DMD*)del45Lumc (MGI:7408338); Tg(DMD*)del51Lumc (MGI:7408340); Tg(DMD*)del53Lumc (MGI:7408339). The animal care and experimental procedures were approved by the Animal Welfare Body Leiden and are in accordance with the Dutch Experiments on Animals Act and EU Directive 2010/63/EU.

### CRISPR/Cas9 editing

Guide RNAs were selected using CRISPOR using the human target region as input sequence, ‘Mus musculus – Mouse – UCSC Jun. 2020 (GRCm39/mm39)’ as reference genome and ‘20bp-NGG – Sp Cas9, SpCas9-HF1, eSpCas9 1.1’ as protospacer adjacent motif. We selected single-guide RNA hits for maximized predicted specificity and predicted efficiency, while discarding any hits with predicted off-targets on mouse chromosome 5 (where the double YAC integration site is found). All CRISPR editing was done using RNP complexes made from Alt-R™ CRISPR recombinant Cas9 and synthetic CRISPR RNA (crRNA) and trans-activating CRISPR RNA (tracrRNA) (IDT) assembled according to the supplier’s protocol. All guide RNA sequences used are provided in Table S1.

### ES culture and blastocyst injection

hDMD/*mdx* ES cells have previously been described (Veltrop et al., 2013). ES cells were cultured in a laboratory utilizing materials that passed the ‘IMPACT 2’ mouse pathogen test (IDEXX BioAnalytix). Cells were regularly checked for mycoplasm infection. To generate Tg(DMD*)del44Lumc, cells were grown on a monolayer of mitotically inactivated mouse embryonic fibroblasts (MEFs) in KnockOut DMEM (Thermo Fisher Scientific) supplemented with 2 mM L-glutamine, 1 mM sodium pyruvate, 1× MEM non-essential amino acid, 0.1 mM 2-mercaptoethanol, 1000 units/ml ESGRO Leukemia Inhibitory Factor (Sigma-Aldrich) and 10% fetal bovine serum and cultured on 0.1% gelatine-coated cell culture plates. For CRISPR-mediated editing of the ES cells, we used two CRISPR RNAs annealed to Alt-R™ CRISPR-Cas9 tracrRNA. The RNP complex was made using 50% Alt-R™ S.p. dCas9 Protein V3 and 50% Alt-R™ S.p. Cas9 Nuclease V3 according to the IDT protocol. The RNP complex was electroporated into the ES cells using a mouse embryonic stem cell nucleofector kit (Lonza) and nucleofector II (Amaxa). Clones were picked into 96-well plates, expanded, and duplicated for freezing and DNA isolation for PCR prescreening. Conditions for PCR prescreening were as follows: 30 s at 95°C; 20 s at 95°C, 20 s at 67°C, 40 s at 72°C for 35 cycles; 1 min at 72°C; using DreamTaq polymerase (Thermo Fisher Scientific). All prescreening primers are listed in Table S2. Primers in intron 43 and intron 44 resulted in a PCR product of ∼500 bp or smaller in the case of a deletion of exon 44 (Fig. 2). Primers in exon 44 and intron 44 were used to check for the presence of exon 44, indicated by the absence of the ∼1600 bp fragment. Copy count by quantitative PCR was done for human *DMD* exons 1, 43, 44, 45 and 79, and chromosomes were counted on candidate clones on metaphase spreads. Correctly targeted ES cells were injected into C57BL/6JrJ mouse blastocysts. The deletion, as determined in the targeted ES cells, was confirmed by ddPCR and Sanger sequencing (Tables S2 and S3). Copy counts for all exons were done on a Bio-Rad QX200 Droplet Digital PCR System according to the protocol provided by the manufacturer. Probes were designed with IDT PrimerQuest™ (Table S3). All assays were done in Bio-Rad ddPCR™ Supermix for Probes (No dUTP) #1863023 with 750 μM of each primer, 250 μM of each probe, and 50 ng DNA purified from ear or tail with a Qiagen DNeasy Blood and Tissue Kit (69504). A single exon probe was amplified in parallel with a reference gene. After droplet generation on the QX200 Droplet Generator, a PCR was performed – 10 min at 95°C; 30 s at 94°C, 1 min at 60°C for 39 cycles; 10 min at 98°C – on a Bio-Rad T100 Thermal Cycler. Droplets were measured on the QX200 Droplet reader, and results were analyzed with QX200 Quantasoft Analysis Pro version 1.7.4.

Chimeric males were bred to C57BL/6Jico/Lumc to establish the Tg(DMD*)del44Lumc (hDMDdel44/*mdx*) mouse line. For Sanger sequencing (in general), the same primers were used as the ones used for PCR. A forward and a reverse read were performed on the PCR product to confirm results, which were analyzed with Snapgene software. Genotyping for the *mdx* exon 23 mutation was performed as previously described (Shin et al., 2011).

### Mouse zygote electroporation

The Tg(DMD*)del45Lumc (hDMDdel45/*mdx*), Tg(DMD*)del51Lumc (hDMDdel51/*mdx*) and Tg(DMD*)del53Lumc (hDMDdel53/*mdx*) lines were generated directly in embryos via electroporation at the zygote stage. For electroporation, a mix of components was prepared in Opti-MEM (31985062, Gibco) including 100 ng/μl ctRNA (tracrRNA and crRNA annealed in IDT duplex buffer), 100 ng/μl SpCas9V3 and 50 ng/μl single-stranded oligodeoxynucleotide (IDT). Freshly obtained fertilized C57BL/6JrJ zygotes were added to the mix, and electroporation was performed using NEPA21 (NEPAGENE) in a CUY501P1-1.5 slide (NEPAGENE), with the following settings: poring pulse (40 V; pulse duration, 3 ms; pulse interval, 50 ms; four pulses) and transfer pulse (5 V; pulse duration, 50 ms; pulse interval, 50 ms; five pulses).

Deletion of both copies of exon 45 or 53 was confirmed in the mosaic offspring using ddPCR copy count and subsequent Sanger sequencing of the deletion-specific prescreen PCR fragments using the same primers as used for amplification. PCR conditions for exon 45 were as follows: 10 min at 95°C; 30 s at 95°C, 30 s at 60°C, 50 s at 72°C for 35 cycles; 5 min at 72°C; using DreamTaq polymerase. PCR conditions for exon 53 were as follows: 5 min at 98°C; 10 s at 98°C, 10 s at 66°C, 20 s at 72°C for 30 cycles; 5 min at 72°C; using Q5 polymerase (Tables S2 and S3). The exon 53 mutant was found to have two different deletions in the copies of the YAC. Here, we cloned the mixed PCR fragment in the pCR4-TOPO TA Vector (Thermo Fisher Scientific) according to the supplied protocol and sequenced 12 clones using the M13F and M13R primers in the vector backbone. For Tg(DMD*)del51Lumc, we only obtained founder animals that had lost one copy of the *hDMD* YAC. Therefore, F1 offspring were subjected to a second round of electroporation with specific guide sequences located within the deleted region, resulting in a mouse line carrying a deletion of both *hDMD* copies, but a slightly different spanning region. Also, for this strain, pups were checked for copy counts by ddPCR and Sanger sequencing as described above (Tables S2 and S3). Furthermore, they were genotyped with the same PCR conditions as those used for the exon 45 PCR. Genotyping for the *mdx* exon 23 mutation was performed as previously described (Shin et al., 2011).

### Histological assessments

Sections of 8 μm thickness were generated of the gastrocnemius of 8-week-old untreated hDMD/*mdx*, C57BL/6J, *mdx*, hDMDdel44/*mdx*, hDMDdel45/*mdx*, hDMDdel51/*mdx* and hDMDdel53/*mdx* male mice. Hematoxylin and Eosin staining was performed to visualize overall histopathology. After defrosting of the slides, they were fixated in ice-cold acetone for 5 min and rinsed with water. Slides were incubated with Hematoxylin for 3 min, and the stain was developed with tap water for 5 min. Thereafter, slides were dipped eight to ten times in acid ethanol and submerged in Eosin for 30 s, followed by incubation in increasing concentrations of ethanol (80, 90, 100%) for 1 min each. After 5 min incubation in xylene, slides were mounted with Pertex, and images were taken at 20× magnification with a BZ-X710 all-in-One Fluorescence Microscope (Keyence, The Netherlands).

The same samples were also stained for dystrophin and laminin. Sections were defrosted and fixated with ice-cold acetone for 5 min, washed for 1 min in PBS and blocked with 1× PBS Tween 20 (0.05%) with 5% horse serum. Slides were incubated with rabbit anti-dystrophin (1:250; ab15277, Abcam; RRID:AB_301813) and rat anti-laminin α2 (1:50; sc-59854, Santa Cruz Biotechnology; RRID:AB_784266), in blocking buffer for 60 min. Thereafter, slides were incubated in blocking buffer with goat-anti-rabbit Alexa Fluor 594 (1:1000; A-11037, Thermo Fisher Scientific, The Netherlands; RRID:AB_2534095) for dystrophin and goat-anti-rat Alexa Fluor 488 (1:1000; A-11006, Thermo Fisher Scientific; RRID:AB_2534074) for laminin. After washing, slides were mounted with ProLong™ Gold Antifade Mountant with DNA Stain DAPI (Thermo Fisher Scientific). Images were taken at 20× magnification using the BZ-X710 all-in-One Fluorescence Microscope (Keyence).

### Western blotting

Muscle sections were homogenized with protein isolation buffer [1.25 mM Tris-HCl pH 6.8, supplemented with 25% (w/v) SDS] for 20 s at speed 7000 two to five times with a MagNA Lyser (Roche Diagnostics) and heated at 95°C for 10 min. Protein concentration was determined with a Bicinchoninic Acid Protein Assay Kit (Thermo Fisher Scientific) according to the manufacturer's instructions. Total protein (30 μg) was added to Laemmli buffer (1.5 M Tris pH 6.8, 10% v/v glycerol, 20% SDS) and β-mercaptoethanol solution and heated at 95°C for 10 min. Samples and a Spectra Multicolor High Range Protein Ladder (Thermo Fisher Scientific) were run on a 3-8% Tris-Acetate gel (Bio-Rad) for 1 h at 75 V and for 1 h at 150 V. Blotting was performed with a Trans-Blot Turbo system (Bio-Rad) with a midi nitrocellulose package at 2.5 A for 10 min. Membranes were incubated with blocking buffer [5 g non-fat dried milk powder (Sigma-Aldrich) in 100 ml 1× Tris-buffered saline (TBS)] for 1 h and washed three times for 15 min with 1× TBS with Tween 20 (TBST). Mouse and human dystrophin protein was detected using the ab154168 primary antibody (1:2000; Abcam; recognizing the last 100 amino acids of the C-terminus; RRID:AB_2858227) in combination with the loading control α-actinin (1:1000; 66895-1, Proteintech; RRID:AB_2882224). Human dystrophin was detected with Mandys106 antibody (1:125; Sigma-Aldrich; Clone2C6, recognizing amino acid 2063-2078; exon 43) in combination with loading control α-actinin (1:1000; ab72592, Abcam; RRID:AB_1524364). TBS incubations were performed in Takara Immuno booster 1 (T7111A, Takara, Göteborg, Sweden) at 4°C overnight. After 3× washing in TBST for 15 min, membranes were incubated with secondary antibodies IRDye 800CW donkey-anti-rabbit (1:5000; Li-Cor) and IRDye 680RD donkey-anti-mouse (1:10,000; Li-Cor), targeting dystrophin and α-actinin, respectively. Membranes were washed in TBST and TBS (both 20 min) and analyzed with an OdysseyCLx imager and software (Li-Cor).

### ASO treatment

ASOs targeting exon 43, 44, 50 or 52 were designed to target regions previously reported by us and others (Aartsma-Rus et al., 2005; Ishizuka et al., 2023) to induce skipping of said exons when developed as 2′-O-methyl phosphorothioate (exon 43, 50 and 52) or phosphorodiamidate morpholino oligomer (exon 44) ASOs (Table S4) and ordered as ViMs (Gene Tools, LLC). ViMs targeted the following exons: exon 43 in the hDMDdel44/*mdx* model, exon 44 in the hDMDdel45/*mdx* model, exon 50 in the hDMDdel51/*mdx* model and exon 52 in the hDMDdel53/*mdx* model. Male mice, aged 8 weeks, received intramuscular injections into the gastrocnemius (50 μl) and triceps (40 μl) with ViMs at a dose of 50 μg (exon 50 and 52 ViMs) or 100 μg (exon 43 and 44 ViMs) per muscle under isoflurane anesthesia on two consecutive days. This difference in dosing was based on the efficiency of the ASOs, where 50 μg of the exon 43 and 44 ViMs did not sufficiently restore dystrophin expression. Animals were sacrificed 2 weeks after the last injection, and muscles were isolated, snap frozen in liquid nitrogen cooled isopentane and stored at −70°C. Sections were generated with a cryotome and divided over two 1.4 mm Zirconium Beads Pre-Filled Tubes (OPS Diagnostics) to allow extraction of both RNA and protein from the same muscle sample.

### RNA isolation and exon skipping analysis

Muscles pieces were homogenized in TRIsure buffer (GC Biotech, The Netherlands) using the MagNA Lyser (Roche Diagnostics). Total RNA was isolated with chloroform in a 1:6 ratio on ice for 5 min. Samples were centrifuged (15,400 ***g*** for 15 min at 4°C), the upper aqueous phase was transferred to a clean tube, and an equal volume of isopropanol was added and precipitated for 30 min. After centrifugation (15,400 ***g*** for 10 min at 4°C), the pellet was washed with 70% ethanol and dissolved in RNase/DNase-free water (Thermo Fisher Scientific). For cDNA synthesis, 1000 ng RNA was added to a mix of deoxynucleotide triphosphates (dNTPs; 10 mM) and random hexamer primers (40 ng/μl), incubated at 70°C for 5 min and chilled on ice. Thereafter, 5× reaction buffer (Promega), RNAsin (40 U/μl, Promega), M-MLV reverse transcriptase (200 U/μl, Promega) and MilliQ were added and left to incubate at 25°C for 10 min, 42°C for 60 min and 70°C for 10 min. Samples injected with saline, minus reverse transcriptase, and water were included as negative controls. Per sample, 2.5 μl cDNA was added to a PCR mixture consisting of 10× DreamTaq buffer (Thermo Fisher Scientific), dNTPs (10 mM), DreamTaq polymerase, MilliQ and ASO-specific primers (ViMs targeting exon 43 or 44: forward primer, 5′-GTCCGTGAAGAAACGATGATG-3′ and reverse primer, 5′-GAGCACTTACAAGCACGGG-3′; ViMs targeting exon 50 or 52: forward primer exon 49, 5′-AAACTGAAATAGCAGTTCAAGC-3′, reverse primer exon 54, 5′-CCAAGAGGCATTGATATTCTC-3′). The PCR consisted of 36 cycles (40 s at 95°C, 40 s at 60°C and 90 s at 72°C). Products were visualized on a 2% agarose gel.

## Supplementary Material

10.1242/dmm.052875_sup1 Supplementary information
