# Supplementary material for: Four new mouse models of Duchenne muscular dystrophy with clinically relevant exon deletions in the human DMD gene
Source: Dis Model Mech. 2026 Aug 28;19(8):dmm052875. doi: 10.1242/dmm.052875 (PMC13580558; doi:10.1242/dmm.052875)
Supplement: Supplementary file 1 [file dmm-19-052875-s1.pdf]

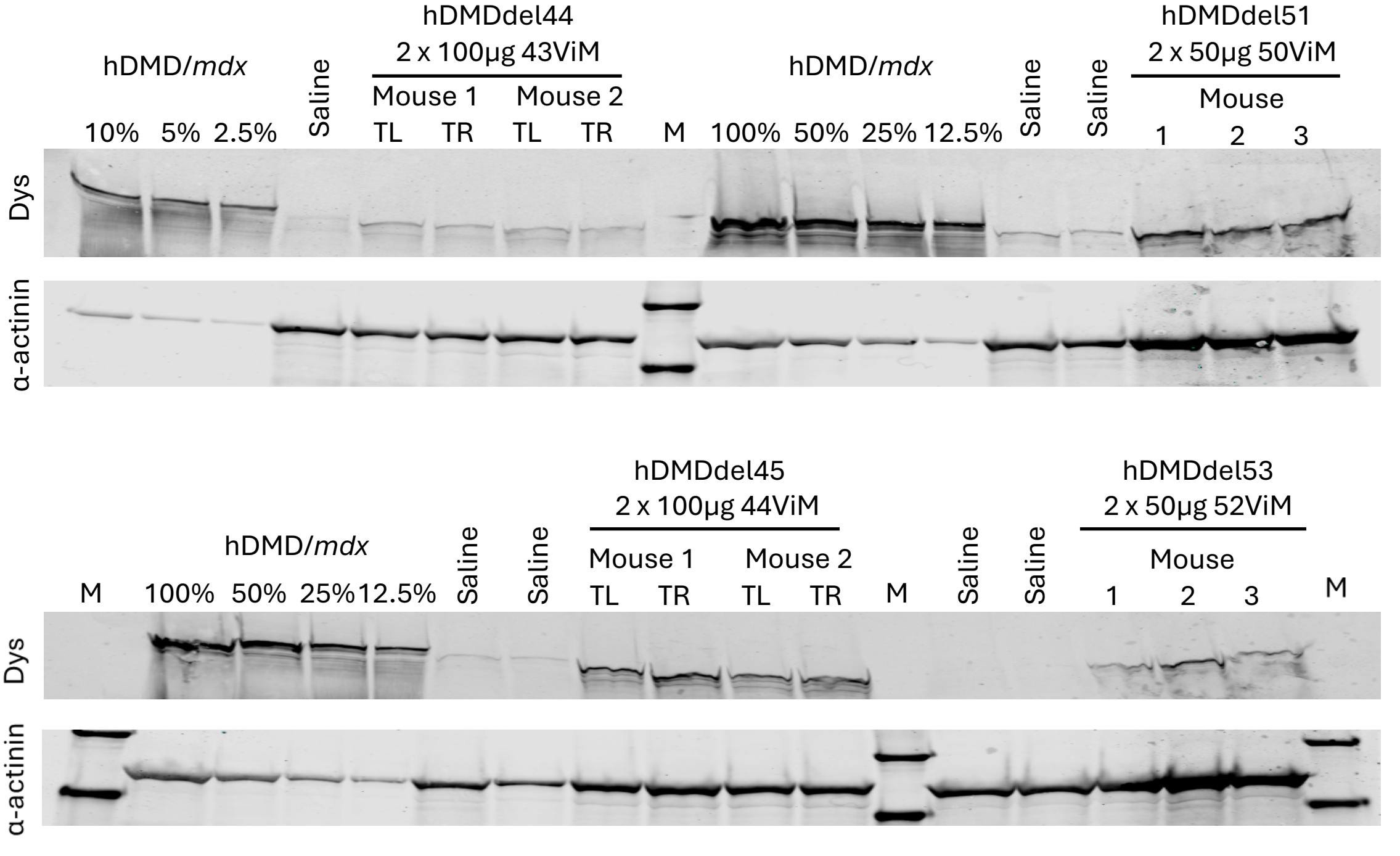

Fig. S1. Dystrophin restoration upon exon skipping in ASO injected triceps samples. Western blot analysis of the triceps utilizing the Ab154168 antibody for dystrophin and the Ab72592 antibody for α-actinin as loading control. Protein was isolated from the right and/or left triceps muscles (respectively TR and TL) of either two or three mice per strain. Dystrophin restoration was observed in all ASO treated muscle samples.

Disease Models & Mechanisms • Supplementary information

Table S1. Details of guide RNAs and resulting deletions

Target exon	Official name	MGI	Sequence sgRNA1	Sequence sgRNA2	Deletion size (nt)	Deletion coordinates
**44**	Tg(DMD*)del44Lumc	7408337	AGCATAAAAAAGGCAACCGA (X:32219068-32219087)	TTTCAATGATATCCAACCCA (X:32215632-32215651)	3584	X:32215512-32219095
**45**	Tg(DMD*)del45Lumc	7408338	TTGCTATTGTGTCAAGGAGT (X:31969558-31969577)	CTATTATGTGGATGATGGGT (X:31967550-31967569)	1994	X:31967567-31969560
**51-1**	Tg(DMD*)del51Lumc	7408339	GTCAGTCAAAAGTCCGTGTG (X:31775395-31775414)	TAGATTTCTAGAGTTCGTGG (X:31772117-31772136)	3277	X:31772132-31775408
**51-2**	Tg(DMD*)del51Lumc	7408339	ACAGCGCCTGACACTATAGT (X:31775251-31775270)	CCAGGACTTTTATTTACCAA (X:31772411-31772430)	2854	X:31772414-31775267
**53-1**	Tg(DMD*)del53Lumc	7408340	TCATTGGAAGTCCAAGAGGG (X:31680756-31680775)	TTAGGAGCTGTCCCCCAACA (X:31678059-31678078)	2720	X:31678059-31680778
**53-2**	Tg(DMD*)del53Lumc	7408340	TCATTGGAAGTCCAAGAGGG (X:31680756-31680775)	TTAGGAGCTGTCCCCCAACA (X:31678059-31678078)	2705	X:31678062-31680769

Table S2. Prescreening primers

Exon		Forward	Sequence	Reverse	Sequence	Size (bp)
44	del	hDMD int43-Fw1	ACTCTTTGACTCCAGTGATGCAA	hDMD int44-Rev	TTCCAAGAAACCCAGCCTGA	318
	wt	hDMD-ex44/Fw1	CCTGCAGGCGATTTGACAGA	hDMD int44-Rev	TTCCAAGAAACCCAGCCTGA	262
45	del	hd_Int44_2_Fw	TCACATGCAACGCTGATCTG	hd_Int45_1_Rev	GTGGGCAAAGACAACTGAC	530
	wt	hd_Int45.1_Fw	TGGCTAGTTAGTGGTTTTCTGC	hd_Int45.1_Rev	GTGGGCAAAGACAACTGAC	700
51-1	del	hd_Int50_2_Fw	GGTGTCCCTGAGAAGCTTGA	hd_Int51_4_Rev	AGTAGAGATGGGGTTTGGCC	880
	wt	hd_Int50.1_fw	AGCCTGGTGTTCTGCAGTTA	hd_int50.1_Rev	TGAGTGGTGAGGGGAAGGAA	730
51-2	del	hd_Int50_2_Fw	GGTGTCCCTGAGAAGCTTGA	hd_Int51_5_Rev	CCTGATGACAAACTGCAAGG	800
51-1 and 51_2	del	hd_Int50.3Fw	CATTCCTTCCCCTCACCACT	hd_Int51.3_Rev	TGGCTTCCCTAAATGCTGGA	300 copy 1<br>730 copy 2
	wt	hd_Int50_4_Fw	GGCATTGTCATACGTGTATTGCT	hd_Int50.4_Rev	CCCTAGGGAAATCAAAGCCAATG	530
53	del	hd_Int52_1_Fw	AGAGGGAGTGCAGTGATCAG	hd_Int53_1_Rev	TGTCCTCATAGCAGCTCAGG	700
	wt	hd_Int52.3_Fw	TGGTGCATTATCAAGCCAGC	hd_ex53.1 Rev	AGACCTGCTCAGCTTCTTCC	757

Disease Models & Mechanisms • Supplementary information

Table S3. ddPCR primers and probes

**Exon**	**Probe**	
**1**	hDMD-ex1/P1	TGTTGGGATCACTCACTTTCCCCC
**6**	hd_Ex6_Probe	TGGCTGGATTGCAACAAACCAACA
**16**	hd_Ex16_Probe	AGCAATCCATGGGCAAACTGTATTCA
**43**	hDMD-ex43/P1	TGCAACGCCTGTGGAAAGGGT
**44**	hDMD-ex44/P1	TCTGTTGAGAAATGGCGGCGT
**45**	hDMD-ex45/P1	TCAGCAATCCTCAAAAACAGATGCCA
**46**	hd_Ex46_Probe	CCACTTGAACCTGGAAAAGAGCAGC
**50**	hd_Ex50_Probe	ACTGGATCCCATTCTCTTTGGCTCT
**51**	hd_Ex51_Probe	CAACGAGATGATCATCAAGCAGAAGGT
**52**	hd_Ex52_Probe	ACAGAGGCGTCCCCAGTTGGA
**53**	HD_Ex53_P2	TCAGAACCGGAGGCAACAGTTGAA
**54**	hd_Ex54_Probe	CTGCAGATGATACCAGAAAAGTCCACA
**58**	hd_Ex58_Probe	ACTCTACCAGGAGCCCAGAGGT
**79**	hDMD-ex79/P1	GGATTTTTCCCGGAGCCGGAA
**Exon**	**Forward**	
**1**	hDMD-ex1/F1	GGCCTCTACAGAATCCTGGC
**6**	hDMD-ex6/F1	GGGTCCGACAATCAACTCG
**16**	hd_Ex16_Fw	TGGAATGCAACCCAGGCTTA
**43**	hDMD-ex43/F1	AGCAAGAAGACAGCAGCATTG
**44**	hDMD-ex44/F1	CCTGCAGGCGATTTGACAGA
**45**	hDMD-ex45/F1	AGAACATTGAATGCAACTGGGG
**46**	hd_Ex46_Fw	TGGTTGGAGGAAGCAGATAACA
**50**	hd_Ex50_Fw	ACCTAGCTCCTGGACTGACC
**51**	hd_Ex51_Fw	GTGATGGTGGGTGACCTTGA
**52**	hd_Ex52_Fw	ACACAACGCTGAAGAACCCT
**53**	HD_ex53/F2	GGGATGAAGTACAAGAACACCT
**54**	hd_Ex54_Fw	TTGGCCCTGAAACTTCTCCG
**58**	hd_Ex58_Fw	CAGAGCAGCCTTTGGAAGGA
**79**	hDMD-ex79/F1	GGCTTACCTGCTTGGTCTAGAAT
**Exon**	**Reverse**	
**1**	hDMD-ex1/R1	GCCTCCCAGATCTGAGTCCT
**6**	hDMD-ex6/R1	CTATGGATGAGAGCATTCAAAGCC
**16**	hd_Ex16_Rev	CATGCTTCCGTCTTCTGGGT
**43**	hDMD-ex43/R1	AGCTGGGAGAGAGCTTCCTG
**44**	hDMD-ex44/R1	TCAGCTTCTGTTAGCCACTGA
**45**	hDMD-ex45/R1	CGCAGATTCAGGCTTCCCAA
**46**	hd_Ex46_Rev	CTTCTTTATGCAAGCAGGCCC
**50**	hd_Ex50_Rev	CTCTCACCCAGTCATCACTTCA
**51**	hd_Ex51_Rev	GTTGCCTAAGAACTGGTGGGA
**52**	hd_Ex52_Rev	TTGGGCAGCGGTAATGAGTT
**53**	HD_ex53/R2	CCTGCTCAGCTTCTTCCTTAG
**54**	hd_Ex54_Rev	CATCCAGTTTCACCACCCCA
**58**	hd_Ex58_Rev	AAGAATGGATGGGCTGCTCC
**79**	hDMD-ex79/R1	AGTGTGGTGTAGTTTCCTCCTGG

Table S4. ViM sequences

**Mouse strain**	**Target exon**	**ViM sequence 5'-3'**
hDMDdel44/*mdx*	43	TTGTTAACTTTTTCCCATTGGAAAT
hDMDdel45/*mdx*	44	TTGTGCTTTCTTCTGTTAGCCAC*
hDMDdel51/*mdx*	50	CCTTCCACTCAGAGCTCAGATCTTC
hDMDdel53/*mdx*	52	GCGGTAATGAGTTCTTCCAACTGGG

* Skipping of exon 44 was done with Brogidirsen. It utilizes 2 linked sequences which target two separate binding sites within exon 44.
